# Tubercular endometritis detected through Pap smear campaign in Enugu, Nigeria

**Published:** 2012-03-15

**Authors:** Wilson Onuigbo, Bessie Esimai, Chinenye Nwaekpe, Grace Chijioke

**Affiliations:** 1 Department of Histopathology, National Orthopaedic Hospital, Enugu, Nigeria; 2 Department Microbiology National Orthopaedic Hospital, Enugu, Nigeria; 3 Medical Women’s Center, Enugu, Nigeria

**Keywords:** Tuberculosis, endometrium, Pap smear, health education, Nigeria

## Abstract

In a series of 3,267 cervical smears examined in Enugu, Nigeria, from 1993 through 2010, there was a single positive case of tuberculosis (TB). It was found in a 55-year-old, Para 7, postmenopausal woman. Treatment for tuberculosis was instituted successfully.

## To the editors of the Pan African Medical Journal

A rare case of tubercular endometritis occurred in a 55-year-old Para 7, postmenopausal woman, who presented herself for cervical smear at the Medical Women’s Centre in Enugu, the capital of Enugu State of Nigeria. The senior author (WO) observed at microscopy the diagnostic Langhans giant cells. This was the only case encountered among 3,267 smears examined between 1993 and 2010. Subsequently, laboratory tests were carried out. The Mantoux test was positive at 14 mm. The ESR measured 52 mm in the first hour. The Total White Count was 7,200/mm^3^. The differential count showed Neutrophils 51%, Lymphocytes 35%, Eosinophils 13%, and Monocytes 1%. Treatment for tuberculosis was instituted and the follow up has been successful.

Pattern of abnormal Pap smears in developing countries is of epidemiological interest. In a Saudi Arabian series [1], there was no mention of tuberculosis. In an Italian study [2], tubercular endometritis was recorded as a rarity which was detected with fluid hysteroscopy. A Chinese study [3] indicated that this disease may cause uterine adhesions, amenorrhea, and subsequently infertility.

A previous report on the easterly Igbos or Ibos [4], one of the largest ethnic groups in Nigeria, led to the following conclusion [5]: “Igbo women conceive and give birth before the onset of genital tuberculous lesions that lead to primary infertility.” The parity of 5 in the instant case is in keeping with this conclusion. Agreeably, as a study [6] centered in the Nigerian middle belt stated, “Endometrial TB is not a frequent cause of infertility in our set-up.”
